# Gradient Estimator-Based Amplitude Estimation for Dynamic Mode Atomic Force Microscopy: Small-Signal Modeling and Tuning

**DOI:** 10.3390/s20092703

**Published:** 2020-05-09

**Authors:** Hafiz Ahmed, Mohamed Benbouzid

**Affiliations:** 1 School of Mechanical, Aerospace and Automotive Engineering, The Futures Institute, Coventry University, Coventry CV1 2TL, UK; hafiz.h.ahmed@ieee.org; 2 Institut de Recherche Dupuy de Lôme (UMR CNRS 6027), University of Brest, 29238 Brest, France; 3 Logistics Engineering College, Shanghai Maritime University, Shanghai 201306, China

**Keywords:** amplitude estimation, gradient estimator, small-signal modeling, atomic force microscopy, sensor signal processing

## Abstract

Atomic force microscopy (AFM) plays an important role in nanoscale imaging application. AFM works by oscillating a microcantilever on the surface of the sample being scanned. In this process, estimating the amplitude of the cantilever deflection signal plays an important role in characterizing the topography of the surface. Existing approaches on this topic either have slow dynamic response e.g., lock-in-amplifier or high computational complexity e.g., Kalman filter. In this context, gradient estimator can be considered as a trade-off between fast dynamic response and high computational complexity. However, no constructive tuning rule is available in the literature for gradient estimator. In this paper, we consider small-signal modeling and tuning of gradient estimator. The proposed approach greatly simplifies the tuning procedure. Numerical simulation and experimental results are provided to demonstrate the suitability of the proposed tuning procedure.

## 1. Introduction

**Figure 1:**
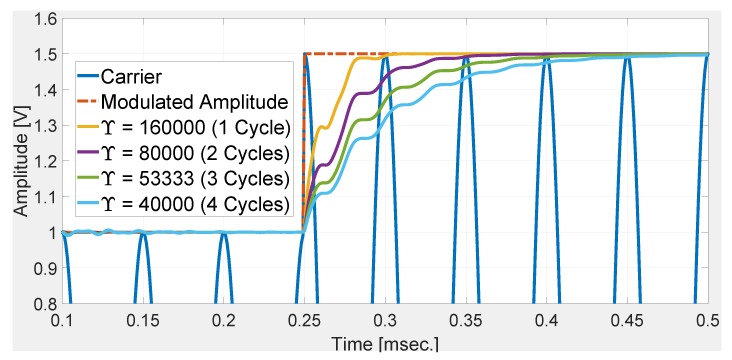
Numerical validation of tuning rule (21).

Atomic force microscopy (AFM) plays an important role in nanoscale imaging in material and biological sciences [1]. Dynamic mode amplitude modulated AFM (AM-AFM) works by forcing a cantilever to oscillate over the surface of the sample being scanned. By estimating the amplitude of cantilever deflection signal, AFM controller makes sure that raster scanning of the sample is performed. An overview of the control-oriented block diagram of AM-AFM can be found in [2] (Figure 1) while details on the working principle are given in [3].

The amplitude estimation part, also known as demodulator, plays an important role in determining the dynamical behavior of AFM, i.e., imaging bandwidth. As a result, fast converging amplitude estimation technique has attracted a lot of attention in the control of AFM research area. One of the basic techniques in this regard is the well known lock-in amplifier (LIA) [4,5,6,7,8,9]. By multiplying the cantilever deflection signal with sine and cosine signals, LIA estimates the amplitude and phase. However, LIA requires high-order low-pass filter (LPF) with high cut-off frequency. As such, the bandwidth is limited by LPFs bandwidth. This problem has been solved in [10] using the idea of orthogonal signal generation. It still requires LPF, however, the cut-off frequency is lower than the standard LIA. LPFs are also used in discrete Fourier transform (DFT)-based LIA proposed in [11]. Some other well known demodulation techniques in the context of AFM are RMS to DC conversion [2], Kalman filter [12,13,14,15], etc. For further investigation on this topic, [16,17] may be consulted as they provide valuable state-of-the-art reviews.

Out of various estimation techniques available in the literature, gradient estimator [18,19,20,21] can be considered as a promising demodulation technique for AFM. By considering the instantaneous estimation error as the cost-function, gradient estimator is obtained through the gradient of the cost-function. This method works by considering a parametric model of the sine wave. Gradient estimator is simple and easy-to-implement. However, to the best of the authors’ knowledge, the tuning procedure of gradient estimator is not straightforward. In general, trial and error [18,19,20] method is used. This is time consuming and application specific.

To overcome the tuning issue of gradient estimator, in this paper, we present a small-signal model of the gradient estimator inspired by [22,23,24]. From the model, simple tuning rule is obtained. The parameter can be easily tuned by selecting the settling time only. This simplifies significantly the tuning of gradient estimator for various practical applications. Through numerical simulation and experimental results, we demonstrate the effectiveness of the proposed tuning method. It is to be noted here that the proposed tuning method can be considered as complimentary to existing results [18,19].

The rest of the paper is organized as follows: Section 2 provides an overview, modeling, and tuning of gradient estimator for amplitude estimation in AM-AFM. Numerical simulation and experimental results are given in Section 3 and finally, Section 4 concludes this paper.

## 2. Gradient Estimator: Small-Signal Modeling and Tuning

A single frequency cantilever deflection signal in the AM-AFM is generally modeled as a sine wave and given as:

(1) y=A(t)sin(ωt+ϕ(t))+ν(t)

where carrier frequency is denoted by ω=2πf, zero-mean measurement noise is denoted by ν(t), instantaneous phase is denoted by ψ=ωt+ϕ(t), and the time-varying amplitude (modulated) and phase are denoted by A(t) and ϕ(t). Model (1) can be extended for multifrequency AFM as:

(2) $$y=\sum_{i=1}^{n}A_{i}\sin\left(\omega_{i}t+\phi_{i}\right)+\nu \left(t\right)$$

where *i* indicates individual component and the time arguments are avoided for notional simplicity. The problem being considered in this paper is to estimate *A* (or $A_{i}$) from the measured cantilever deflection signal (1) (or (2)). This will be achieved using gradient approach. For further development, model (1) can be written in the linear parametric form as:

(3) $$y=\Phi^{T}\Theta .$$

where $\Phi ={\left[\sin\left(\omega t\right)\;\cos\left(\omega t\right)\right]}^{T}$ and $\Theta ={\left[A\cos\left(\phi \right)\;A\sin\left(\phi \right)\right]}^{T}$. From the parameter vector Θ, the instantaneous amplitude and phase can be calculated as:

(4) $$\begin{array}{cc} A & =\sqrt{\Theta^{T}\Theta }, \end{array}$$

(5) $$\begin{array}{cc} \phi & =\mathrm{atan}2\left(\Theta_{2},\Theta_{1}\right) \end{array}$$

### 2.1. Brief Overview of the Gradient Estimator

To estimate the parameters from the measured signal *y*, let us consider the following quadratic cost-function [21]:

(6) $$J\left(\hat{\Theta }\right)=\frac{e^{T}e}{2}$$

where $e=\left(y-\Phi^{T}\hat{\Theta }\right)$ and ^ represents estimated value. Gradient estimator is generally designed by minimizing the cost-function (6). The solution of $\hat{\Theta }$ that minimizes the cost-function (6) is generated by [21]:

(7) $$\dot{\hat{\Theta }}=-\Omega \Delta J\left(\hat{\Theta }\right)$$

where $\Omega =\Omega^{T}=YI_{2},Y>0$ is the gain matrix with Y being the tuning parameter. From Equation (6), gradient of the cost-function can be obtained as [21], Appendix B.2 of [25]:

(8) $$\Delta J\left(\hat{\Theta }\right)=\frac{\delta J}{\delta e}\frac{\delta e}{\delta \hat{\Theta }}=-\Phi^{T}e$$

By substituting Equation (8) into (7), the gradient estimator can be obtained as:

(9) $$\begin{array}{cc} {\dot{\hat{\Theta }}}_{1} & =Y\sin(\omega t)e, \end{array}$$

(10) $$\begin{array}{cc} {\dot{\hat{\Theta }}}_{2} & =Y\cos(\omega t)e \end{array}$$

Convergence speed of the gradient estimator is controlled by the tuning parameter Y.

### 2.2. Small-Signal Modeling

For the modeling purpose, in this section, we assume that $A\approx \hat{A}$, $\phi \approx \hat{\phi }$, and $\psi \approx \hat{\psi }$. Moreover, small-angle approximation formulas will be used i.e., sin(ψ)≈ψ and cos(ψ)≈1. The estimated amplitude and its dynamics are given by:

(11) $$\begin{array}{cc} \hat{A} & =\sqrt{\Theta^{T}\Theta }=\sqrt{{\hat{\Theta }}_{1}^{2}+{\hat{\Theta }}_{2}^{2}}, \end{array}$$

(12) $$\begin{array}{cc} \dot{\hat{A}} & =\left({\hat{\Theta }}_{1}{\dot{\hat{\Theta }}}_{1}+{\hat{\Theta }}_{2}{\dot{\hat{\Theta }}}_{2}\right)/\left(\sqrt{{\hat{\Theta }}_{1}^{2}+{\hat{\Theta }}_{2}^{2}}\right) \end{array}$$

By substituting the value of ${\hat{\Theta }}_{1}$$=\hat{A}\cos\left(\hat{\phi }\right)$ and ${\hat{\Theta }}_{2}=\hat{A}\sin\left(\hat{\phi }\right)$ in Equation (12), one can obtain:

(13) $$\dot{\hat{A}}=\frac{Y\left\{\hat{A}\cos\left(\hat{\phi }\right)\sin\left(\omega t\right)+\hat{A}\sin\left(\hat{\phi }\right)\cos\left(\omega t\right)\right\}e}{\hat{A}}$$

By substituting $\sin\left(\omega t\right)\cos\left(\hat{\phi }\right)+\cos\left(\omega t\right)\sin\left(\hat{\phi }\right)=\sin\left(\omega t+\hat{\phi }\right)=\cos\left(\hat{\psi }\right)$ and $e=A\sin\left(\psi \right)-\hat{A}\sin\left(\hat{\psi }\right)$ in Equation (13), the following can be obtained:

(14) $$\begin{array}{cc} \dot{\hat{A}} & =\frac{Y\hat{A}\sin\left(\hat{\psi }\right)\left\{A\sin\left(\psi \right)-\hat{A}\sin\left(\hat{\psi }\right)\right\}}{\hat{A}}\\ \; & =\frac{Y\left\{\underset{A\cos\left(\psi -\hat{\psi }\right)-A\cos\left(\psi +\hat{\psi }\right)}{\underset{︸}{2A\sin\left(\psi \right)\sin\left(\hat{\psi }\right)}}-\underset{\underset{\hat{A}\cos\left(2\hat{\psi }\right)-\hat{A}}{\underset{︸}{2\hat{A}\left\{\cos^{2}\left(\hat{\psi }\right)-1\right\}}}}{\underset{︸}{2\hat{A}\sin^{2}\left(\hat{\psi }\right)}}\right\}}{2}\\ \; & =\frac{Y\left\{A\underset{\approx 1}{\underset{︸}{\cos\left(\psi -\hat{\psi }\right)}}\underset{\approx 0}{\underset{︸}{-A\cos\left(\psi +\hat{\psi }\right)+\hat{A}\cos\left(2\hat{\psi }\right)}}-\hat{A}\right\}}{2}\\ \dot{\hat{A}} & \approx \frac{Y}{2}\left(A-\hat{A}\right) \end{array}$$

Similarly, the estimated phase and its dynamics are given by:

(15) $$\begin{array}{cc} \hat{\phi } & =\mathrm{atan}2\left({\hat{\Theta }}_{2},{\hat{\Theta }}_{1}\right), \end{array}$$

(16) $$\begin{array}{cc} \dot{\hat{\phi }} & =\left({\hat{\Theta }}_{1}{\dot{\hat{\Theta }}}_{2}-{\hat{\Theta }}_{2}{\dot{\hat{\Theta }}}_{1}\right)/\left({\hat{\Theta }}_{1}^{2}+{\hat{\Theta }}_{2}^{2}\right) \end{array}$$

By substituting the value of ${\hat{\Theta }}_{1}=\hat{A}\cos\left(\hat{\phi }\right)$, ${\hat{\Theta }}_{2}=\hat{A}\sin\left(\hat{\phi }\right)$ in Equation (16), one can obtain:

(17) $$\dot{\hat{\phi }}=\frac{Y\left\{\hat{A}\cos\left(\hat{\phi }\right)\cos\left(\omega t\right)-\hat{A}\sin\left(\hat{\phi }\right)\sin\left(\omega t\right)\right\}e}{{\hat{A}}^{2}}$$

By substituting $\cos\left(\hat{\phi }\right)\cos\left(\omega t\right)-\sin\left(\hat{\phi }\right)\sin\left(\omega t\right)=\cos\left(\omega t+\hat{\phi }\right)=\cos\left(\hat{\psi }\right)$ and $e=A\sin\left(\psi \right)-\hat{A}\sin\left(\hat{\psi }\right)$ in Equation (17), the following can be obtained:

(18) $$\begin{array}{cc} \dot{\hat{\phi }} & =\frac{Y\cos\left(\hat{\psi }\right)\left\{A\sin\left(\psi \right)-\hat{A}\sin\left(\hat{\psi }\right)\right\}}{\hat{A}}\\ \; & =\frac{Y\left\{\underset{A\sin\left(\psi +\hat{\psi }\right)+A\sin\left(\psi -\hat{\psi }\right)}{\underset{︸}{2A\cos\left(\hat{\psi }\right)\sin\left(\psi \right)}}-\underset{\hat{A}\sin\left(2\hat{\psi }\right)}{\underset{︸}{2\hat{A}\cos\left(\hat{\psi }\right)\sin\left(\hat{\psi }\right)}}\right\}}{2\hat{A}}\\ \; & =\frac{Y\left\{\underset{\approx 0}{\underset{︸}{\underset{\approx \hat{A}\sin\left(2\hat{\psi }\right)}{\underset{︸}{A\sin\left(\psi +\hat{\psi }\right)}}-\hat{A}\sin\left(2\hat{\psi }\right)}}+\underset{\approx \hat{A}\left(\psi -\hat{\psi }\right)}{\underset{︸}{A\sin\left(\psi -\hat{\psi }\right)}}\right\}}{2\hat{A}}\\ \dot{\hat{\phi }} & \approx \frac{Y}{2}\left(\psi -\hat{\psi }\right)\\ \dot{\hat{\phi }} & \approx \frac{Y}{2}\left(\phi -\hat{\phi }\right) \end{array}$$

From Equation (14) and (18), transfer function of the estimated amplitude and phase can be found as:

(19) $$\frac{\hat{\phi }}{\phi }\left(s\right)=\frac{\hat{A}}{A}\left(s\right)=G\left(s\right)=\frac{\frac{Y}{2}}{s+\frac{Y}{2}}$$

where zero initial conditions are assumed. From model (19), it can be seen that the gradient estimator has a first-order dynamics, at least locally.

### 2.3. Parameter Tuning

To tune the gradient estimator parameter Y, let us rewrite the transfer function G(s) as:

(20) $$G\left(s\right)=\frac{1}{\tau s+1}$$

where τ=2/Y is the time constant. It is well known that for a first-order system, the settling time is given by $t_{ss}=4\tau$. Then by using the value of τ, the formula to tune the estimator gain Y is given by:

(21) $$Y=\frac{8}{4\tau }=\frac{8}{t_{ss}}.$$

To validate the tuning rule (21), let us consider a step change in the modulated amplitude with a carrier signal of 20 kHz. Four different tuning gains have been considered. They are Y=16000,8000,53333,4000 and correspond to a settling time of 1,2,3, and 4 cycles, respectively. Numerical simulation results are given in Figure 1. Results in this figure show that the estimated amplitudes converged roughly within the desired settling times. This shows the suitability of the proposed tuning method.

### 2.4. Extension to Harmonic Deflection Signal

For the sake of computational simplicity, gradient estimator was designed in Section 2.1 only by considering the fundamental component. However, it can be easily extended to deflection sensor signal with arbitrary order harmonics. In this case, by considering the multifrequency deflection signal (2), linear parametric model (3) can be rewritten as:

(22) $$y=\Phi^{T}\Theta$$

where the information and parameter vector are given as:

(23) $$\begin{array}{cc} \Phi & ={\left[\begin{array}{ccccccc} \sin(\omega t) & \cos(\omega t) & \sin(2\omega t) & \cos(2\omega t) & \ldots & \sin\left(n\omega t\right) & \cos(n\omega t) \end{array}\right]}^{T}, \end{array}$$

(24) $$\begin{array}{cc} \Theta & ={\left[\begin{array}{ccccccc} A_{1}\cos\left(\phi_{1}\right) & A_{1}\sin\left(\phi_{1}\right) & A_{2}\cos\left(\phi_{2}\right) & A_{2}\sin\left(\phi_{2}\right) & \ldots & A_{n}\cos\left(\phi_{n}\right) & A_{n}\sin\left(\phi_{n}\right) \end{array}\right]}^{T}. \end{array}$$

Then, the gradient estimator for the signal (22) in vector form is given by:

(25) $$\dot{\hat{\Theta }}=\Omega \Phi^{T}e$$

where $e=y-\Phi^{T}\Theta$ is the estimation error and $\Omega =\Omega^{T}=YI_{2n},Y>0$ with $I_{2n}$ being the identity matrix of dimension 2n×2n. Alternatively, the gradient estimator (25) can be implemented in parallel form as given in [19]. This can help to reduce the computational complexity thanks to parallel implementation. A similar parallel approach has also been used in grid-synchronization literature [26].

## 3. Results and Discussions

### 3.1. Simulation Study

Karvinen and Moheimani [10] showed that signal (26) can model the response of Bruker DMASP microcantilever signal.

(26) $$y\left(t\right)=A\left(t\right)\cos\left(\omega t\right)+0.1\cos\left(4\omega t+\phi \right)+\nu \left(t\right)$$

where $A\left(t\right)=1+0.1\mathrm{sgn}(\sin\left(2\pi f_{m}t\right))$, where sgn(.) is the signum function and $f_{m}$ is the amplitude modulation frequency. For the simulation study, we have considered *f* = 20 kHz, $f_{m}$ = 1 kHz, and ϕ=π/4.

To tune the gradient estimator (GE) presented in Section 2.4 for signal (26), we will consider the tuning formula (21). Let us consider a 2-cycle settling time i.e., $t_{ss}=10^{-4}$ s. Then, the gain of the gradient estimator can be found as Y=80,000. As comparison techniques, high bandwidth demodulation (HBD) technique [10] and Kalman filter (KF) have been selected. Third-order Butterworth low-pass filters with cut-off frequency of 4 kHz haven been considered for HBD technique. Parameters of Kalman filter are selected as: R=1, Q=0.05, and *P*$=1000I_{4}$, where $I_{2}$ is the identity matrix of dimension 4×4. All the techniques have been implemented in Matlab/Simulink with a sampling frequency of 200 kHz. Continuous integrators of gradient estimator’s are discretized using Euler method, i.e., ODE1.

**Figure 2:**
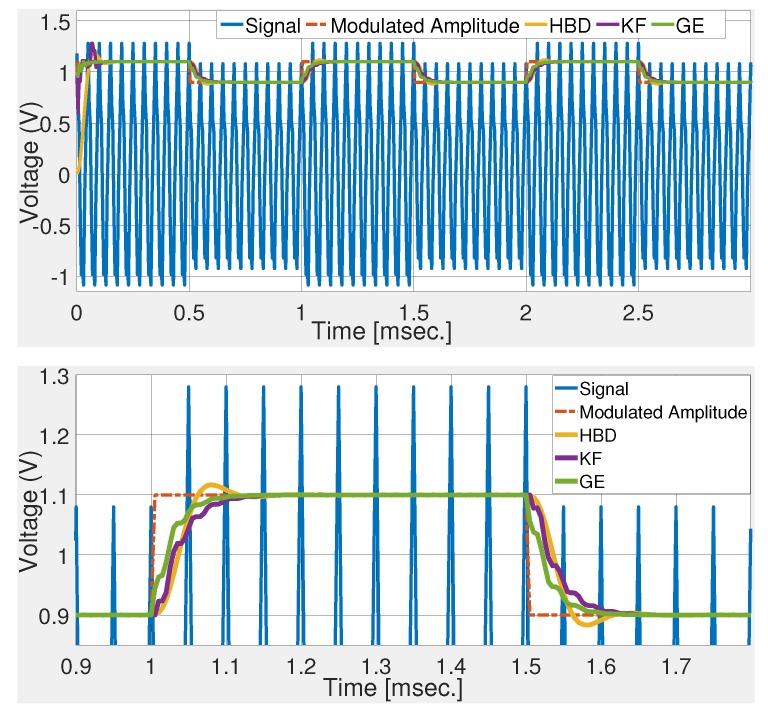
Comparative numerical simulation results with microcantilever signal (26).

**Figure 3:**
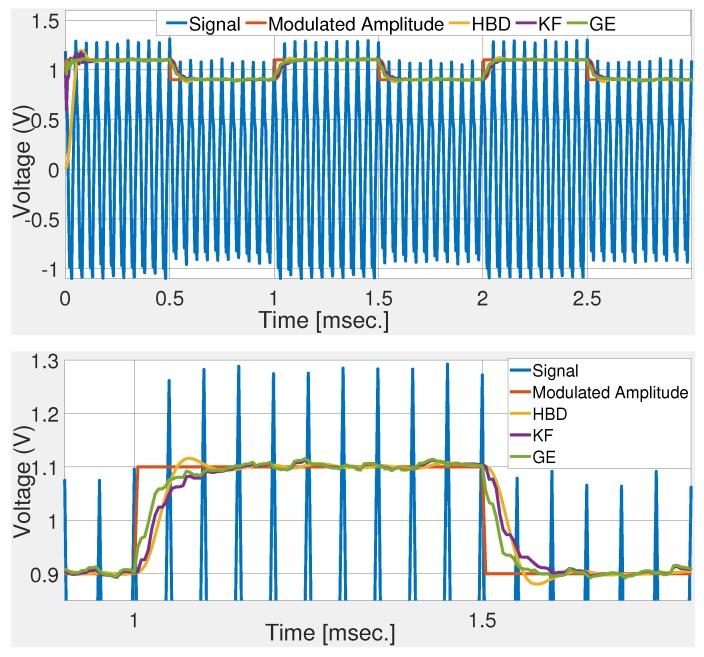
Comparative numerical simulation results with noisy deflection sensor signal (signal-to-noise ratio—SNR of 34 dB).

**Figure 4:**
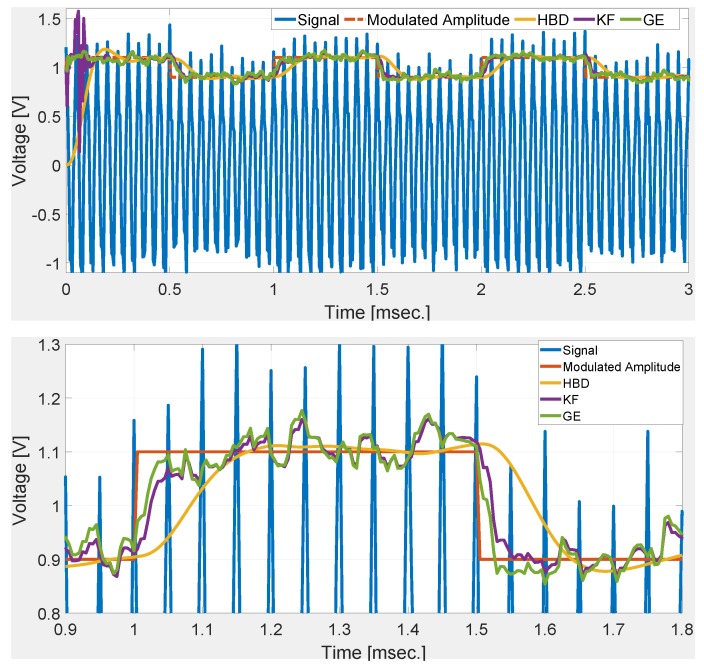
Comparative numerical simulation results with noisy deflection sensor signal (SNR of 20 dB).

Figure 2 shows the comparative performance of the selected techniques. Numerical simulation results show that the gradient technique roughly converged in 2 cycles. This validates the control parameter tuning. Simulation results show that GE has the fastest rise and settling time. KF and GE has a first-order response while HBD shows a second-order response with overshoot. Simulation results shown in Figure 2 demonstrate the suitability of the GE over KF and HBD in noise-free condition. To test the noise robustness of the comparative techniques, band-limited white noise is added to the deflection signal. Numerical simulation results with signal-to-noise ratio (SNR) of 34 dB and 20 dB are given in Figure 3 and Figure 4, respectively. These figures show that KF and GE have similar noise robustness while HBD performs slightly better in the steady-state. This is possible due to the presence of two third-order LPFs. However, this also slows down the dynamic response for HBD. Moreover, from Figure 4, it can be seen that HBD never really converged.

**Figure 5:**
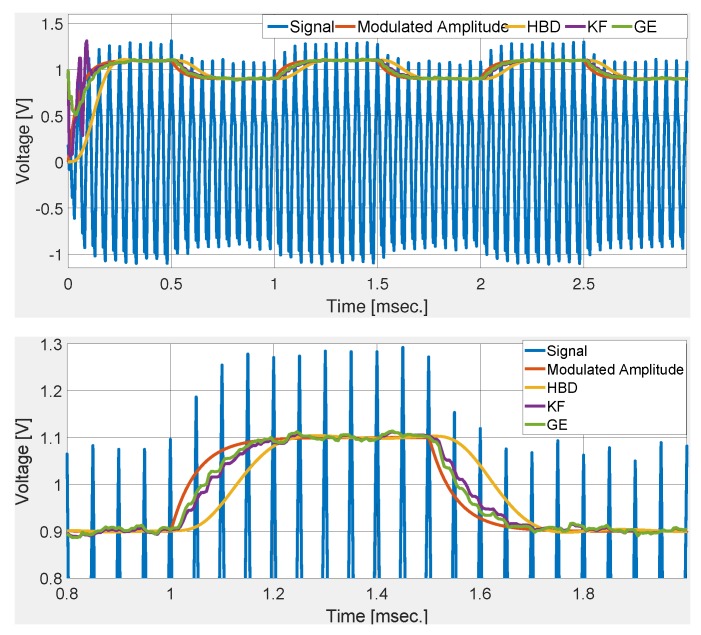
Comparative numerical simulation results with gradual change in the modulated amplitude and SNR of 34 dB.

**Figure 6:**
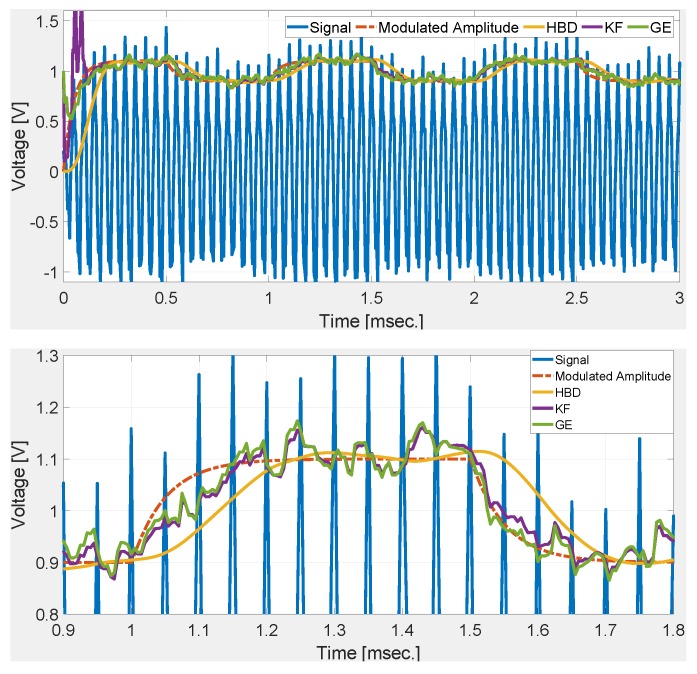
Comparative numerical simulation results with gradual change in the modulated amplitude and SNR of 20 dB.

In the previous two cases, step change in the modulated amplitude is considered. However, in practice, gradual change in amplitude may also be observed. To simulate this situation, modulated amplitude A(t) is passed through a first-order low-pass filter with cut-off frequency 20kHz. Numerical simulation results with SNR of 34 dB and 20 dB are given in Figure 5 and Figure 6, respectively. Comparative results show that GE most closely follows the gradually changing modulated amplitude followed by KF. HBD has a significant delay compared to GE and KF which is largely attributed to the presence of LPFs. Simulation results in Figure 5 show that the GE is not only suitable for step change but also for gradual change in the modulated amplitude. This makes the proposed tuning method highly suitable for practical implementation in real AFM system.

### 3.2. Experimental Study

**Figure 7:**
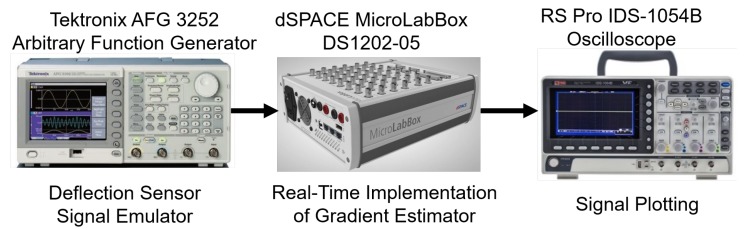
Overview of the experimental setup.

dSPACE-based experimental study is considered in this section [27]. The considered experimental setup is given in Figure 7. In this setup, an arbitrary function generator (Tektronix AFG 3252) is used to generate the emulated deflection sensor signal. This analog signal is acquired through the input/Output board DS 1302-03 available in dSPACE MicroLabBox (DS 1202-05). MicroLabBox also hosts the real-time implementation of the comparative techniques. Finally, a digital storage oscilloscope (RS Pro IDS-1054B) is used to plot the outputs. For experimental implementation, frequency of the deflection sensor signal is considered as 5 kHz while the amplitude modulation frequency was 0.5 kHz. Sampling frequency for the real-time implementation was 50 kHz.

**Figure 8:**
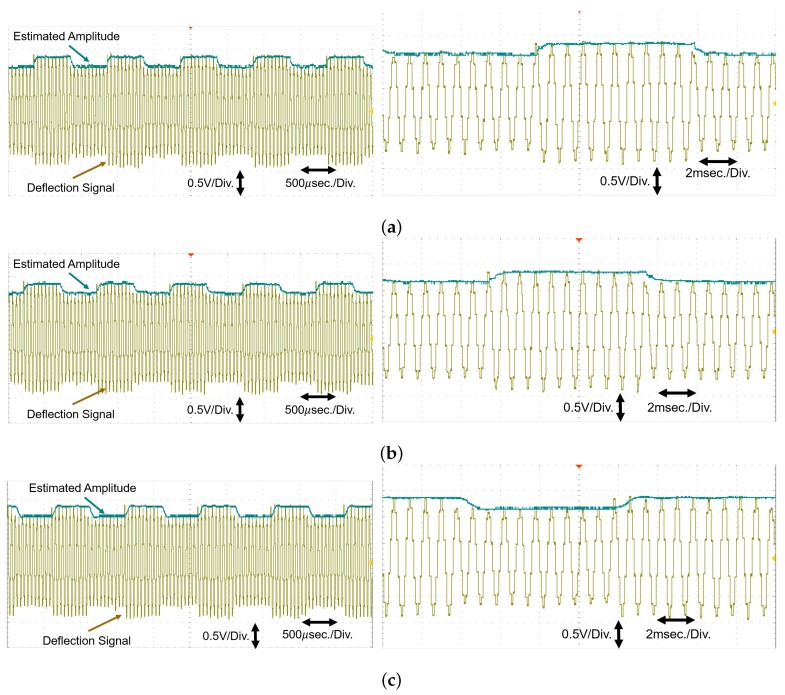
Comparative experimental results using setup in Figure 7: (**a**) Gradient estimator (left—original view, right—zoomed view), (**b**) Kalman Filter (left—original view, right—zoomed view), and (**c**) High bandwidth demodulation (left—original view, right—zoomed view).

Comparative experimental results are given in Figure 8. Figure 8 shows that the experimental results are similar to the numerical simulation results. All the techniques have similar steady-state performance. However, the gradient estimator has the fastest rise and settling time. It is computationally simpler than Kalman filter and low-pass filtering free unlike HBD. Experimental results show the suitability of the gradient estimator as an amplitude demodulation technique for dynamic mode amplitude modulated atomic force microscopy. Experimental results also validate the tuning rule developed in this paper in Section 2.3.

## 4. Conclusions and Future Work

This paper has proposed small-signal modeling and tuning of gradient estimator for amplitude estimation of deflection signal used in dynamic mode amplitude modulated atomic force microscopy. Small-signal model can facilitate quick tuning of gradient estimator parameter. The developed model and tuning rule were validated through numerical simulation and experimental results. Comparative results validated the performance of the proposed tuning procedure with two other advanced techniques. In the current work, an instantaneous cost-function was considered for the gradient estimator design. To enhance the noise robustness property, discount integral cost-function will be considered in a future work.
